# Signalment and Clinical Data of Cats with Exocrine Pancreatic Insufficiency Diagnosed Using Feline Trypsin-like Immunoreactivity in Routine Diagnostics

**DOI:** 10.3390/vetsci8080155

**Published:** 2021-08-03

**Authors:** Katrin Törner, Julia Maria Grassinger, Corinna N. Weber, Heike Aupperle-Lellbach, Argine Cerezo-Echevarria, Elisabeth Müller

**Affiliations:** LABOKLIN GmbH & Co. KG, 97688 Bad Kissingen, Germany; grassinger@laboklin.de (J.M.G.); weber@laboklin.de (C.N.W.); aupperle@laboklin.de (H.A.-L.); cerezo@laboklin.de (A.C.-E.); mueller@laboklin.de (E.M.)

**Keywords:** fTLI, ELISA, EPI, cat, signalment, clinical data, reference interval

## Abstract

Serum feline trypsin-like immunoreactivity (fTLI) is commonly used to diagnose feline exocrine pancreatic insufficiency (EPI). This study aimed to describe signalment and clinical data of cats with EPI. Determination of TLI was performed using an in-house ELISA; the reference interval was defined using a Reference Limit Estimator. Groups were formed from 4813 cats (2019–2020), based on their fTLI concentration: 1 (<8 µg/L; decreased; *n* = 275), 2 (8–88 µg/L; reference interval; *n* = 4256), and 3 (>88 µg/L; increased; *n* = 282). Males and Domestic Shorthairs were most common in all groups. Group 3 had the highest (13 years), and group 1 had the lowest (9 years), median age. Clinical information was available for 200 cats (decreased fTLI: *n* = 87, lower reference interval (8–12 µg/L): *n* = 113). Treatment response was observed in 83% (decreased fTLI) and 66% (lower reference interval). EPI cats displayed weight loss (69%), diarrhoea (68%), vomiting (41%), anorexia (39%), poor hair coat (35%), lethargy (33%), and/or polyphagia (21%). The lower the serum fTLI concentration, the more often good treatment response was reported (*p* = 0.022) but there were no statistically significant clinical signs. In conclusion, fTLI is a helpful parameter to diagnose EPI but predicting treatment response based on signalment or clinical signs is not possible.

## 1. Introduction

Feline exocrine pancreatic insufficiency (EPI) is a rare disease caused by the lack of synthesis and/or secretion of pancreatic enzymes, which leads to intestinal malabsorption [1]. Prevalence in the United States is between 0.013% and 0.103% [2]. However, canine EPI is well described, especially in the German Shepherd breed [3]. In dogs, the cause for this is most often associated with pancreatic acinar atrophy, while chronic pancreatitis is rarely reported [4,5]. In contrast, severe chronic pancreatitis with subsequent extensive tissue destruction (atrophy, fibrosis, and loss of the acinar cells) is thought to be the most common cause of EPI in cats [6].

Generally, cats with EPI have more non-specific clinical signs than dogs. Most cats display weight loss and unformed faeces (occasionally diarrhoea); poor hair coat, anorexia, increased appetite, and/or lethargy were reported irregularly [6,7] thus often making it challenging to diagnose EPI.

Measuring trypsin and trypsinogen, serum feline trypsin-like immunoreactivity (fTLI) is a specific assay to evaluate the capacity of the exocrine pancreas and is therefore specific for the diagnosis of EPI in cats [6]. Different methods were used for this test, including a radioimmunoassay (RIA) [8] and an enzyme-linked immunosorbent assay (ELISA) [9]. Species-specific tests have been described for dogs [10] and recently, for ferrets as well [11]. However, even though feline EPI is now diagnosed more often, it is likely still underdiagnosed given the non-specific clinical features [1], and substantial data on diagnosed cases is limited. Cats with uncomplicated EPI have a good prognosis after adequate treatment [2]. However, individual critical cases, such as respiratory failure secondary to severe hypokalaemia [12] or vitamin K-responsive coagulopathy [13] due to malabsorption, are described as well.

Most cats with EPI can be successfully managed by dietary supplementation of pancreatic enzymes (dried extracts or raw porcine pancreas) [1]. As hypocobalaminemia is common in cats with EPI [14] cobalamin should be measured in all cats with suspected EPI and supplemented if needed [1]. In addition, cats diagnosed with diabetes mellitus due to chronic pancreatitis are at high risk of developing EPI, which should be considered as a subsequent, possible complication [15].

This study aimed to investigate the signalment of cats with suspected EPI and to evaluate signalment, clinical signs, and treatment response of cats diagnosed with EPI based on a low serum fTLI concentration.

## 2. Materials and Methods

### 2.1. Serum fTLI Concentration

Serum fTLI concentration was quantified by an in-house sandwich ELISA using antibodies against feline trypsinogen. Antibodies were produced using native feline trypsinogen isolated from the pancreas of cats. In short, serum samples were diluted 1:4 with a buffer and added to a microplate coated with antibodies against feline TLI. Microplate wells were incubated at 37 °C with a Horseradish Peroxidase (HRP) conjugate solution containing anti-feline antibodies. After washing them four times, 3,3′,5,5′-Tetramethylbenzidine (TMB) substrate solution was added. The reaction was stopped after 15 min with 1 N H2SO4. Samples were measured immediately after stopping, using an absorbance-based microplate reader at 450 nm against a standard dilution of native feline trypsinogen. The intra- and inter-assay variation coefficients of the in-house feline trypsin-like immunoreactivity ELISA were 2.34% and 5.15%, respectively. The limit of detection (LOD) was established at 0.5 µg/L, and the standard dilutions ranged between 3.25 and 104 µg/L.

For this study, reference intervals were calculated from samples from 2019–2020 using the Reference Limit Estimator (version RLE49 20180517, Deutsche Gesellschaft für Klinische Chemie und Laboratoriumsmedizin e.V., Bonn, Germany) with R version 4.0.5. This method uses an algorithm to exclude presumably non-healthy individuals by decreasing the statistical error based on a high number of data sets, and, therefore, knowledge of the health status is not necessary [16]. For statistical analysis, serum fTLI values <0.1 µg/L were replaced with 0.09 µg/L, and values >120 µg/L with 120.1 µg/L.

To assess the representability of the obtained data, a larger population from 2017–2020 was used for calculation additionally. Signalment for the latter one (2017–2020) was incomplete. All data sets were exported to Excel spreadsheets for further processing.

### 2.2. Signalment and Clinical Data

Data sets from 4813 serum samples with complete signalment (breed, sex, and age) submitted to LABOKLIN GmbH & Co. KG from 2019–2020 for routine fTLI measurement were reviewed retrospectively. For statistical analysis, format and nomenclature were standardised in Excel. Three groups were created based on the serum fTLI concentration:

- Group 1 (serum fTLI < 8 µg/L, decreased).
- Group 2 (serum fTLI 8–88 µg/L, reference interval).
- Group 3 (serum fTLI > 88 µg/L, increased).

In the next step, clinical data were requested from the submitting veterinarians for cats with serum fTLI concentrations <8 µg/L and cats with serum fTLI within the lower reference interval of 8–12 µg/L. This cut-off was set based on the previously established reference intervals for the in-house ELISA which included a grey zone of 8–12 µg/L (unpublished data). The additional group with values of 8–12 µg/L was evaluated separately to take a possible early-stage EPI into account. Feline serum samples sent from clinicians for initial serum fTLI determination were included, with follow-up examinations being discarded. The veterinarians were asked to provide information regarding clinical signs, previous history, treatment, and subsequent response or lack of response to treatment (yes or no). It was not possible to obtain a complete set of clinical data from all animals, mainly because the submitting veterinarian’s documentation was incomplete or there was no follow-up. Those cats for which clinical data was available were divided into two subgroups based on the serum fTLI concentration:

- Group 1.1 (serum fTLI <8 µg/L and clinical data available).
- Group 2.1 (serum fTLI 8–12 µg/L and clinical data available).

Both groups were then further subdivided into “responsive” (groups 1.1a and 2.1a) and “non-responsive” (groups 1.1b and 2.1b) to pancreatic enzyme supplementation. Treatment response was defined as the noticeable improvement of the clinical signs including weight gain, improved stool consistency, vomiting resolution, normalisation of food intake, and/or general condition after pancreatic enzyme supplementation.

Statistical analysis was performed using the software SPSS for Windows (version 26; IBM, Armonk, NY, USA). The Kolmogorov-Smirnov and Shapiro-Will tests were used to test the data for normal distribution. Moreover, the Mann-Whitney-U, Kruskal-Wallis, and Pearson and Spearman Rank correlation tests were performed. *p* values < 0.05 were considered statistically significant.

## 3. Results

### 3.1. Re-Evaluation of Reference Interval

**Figure 1:**
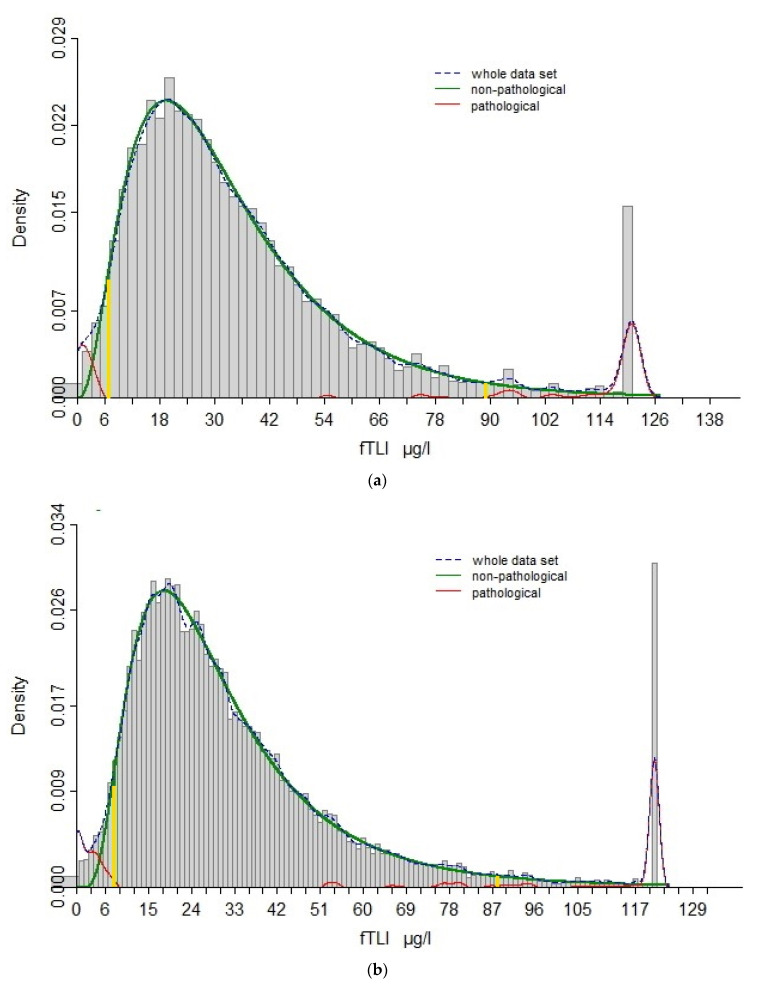
Feline trypsin-like immunoreactivity interval based on (**a**): 4813 cats (2019–2020) and (**b**): 21,714 cats (2017–2020), calculated using the Reference Limit Estimator with an algorithm to exclude presumably non-healthy individuals by decreasing the statistical error based on a high number of data sets. The *x*-axis represents the serum fTLI concentrations and the *y*-axis gives the density/frequency of the values. The dotted blue line represents all data sets. This is further divided mathematically into non-pathological (green line) and pathological (red line) values. The vertical yellow lines represent the 2.5 (**a**: 7 µg/L, **b**: 8 µg/L) and 97.5 (**a**: 89 µg/L, **b**: 88 µg/L) percentiles of the estimated distribution for non-pathological values. As it is a mathematical formula/algorithm, little overlap is expected in both directions, with few non-pathological serum fTLI concentrations within the estimated reference limits, and few pathological ones below or above the reference limits.

Between 2019 and 2020, serum fTLI concentration was routinely measured from 4813 feline samples. The serum fTLI reference interval was established between 7 µg/L and 89 µg/L by the Reference Limit Estimator (Figure 1a).

To test representability, the reference limits were estimated according to a larger cohort, consisting of all cases submitted between 2017 and 2020 (*n* = 21,714). The results displayed good agreement (Figure 1b), with the lower and upper reference limits at 8 µg/L and 88 µg/L, respectively. The reference interval 8–88 µg/L obtained with the larger group was used for the ongoing study, and the subgroup of 4813 cats was considered as representative for further assessment in this study.

### 3.2. Signalment and Clinical Data

**Figure 2:**
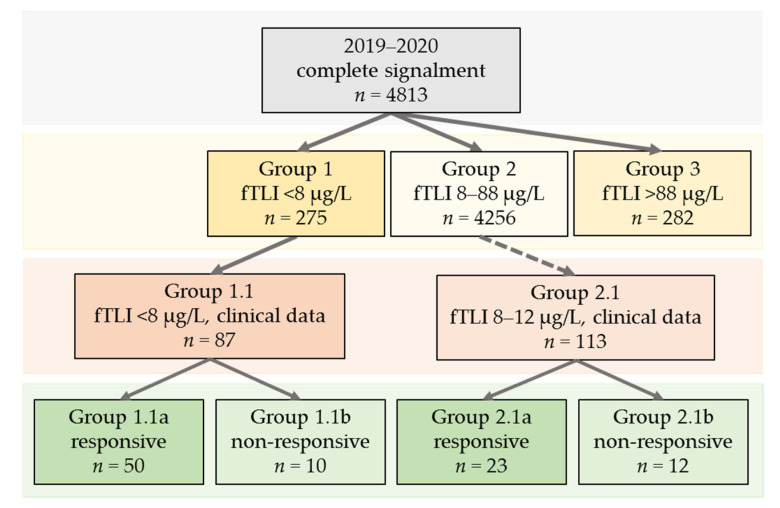
Overview of the analysed data sets and groups. The cases from 2019–2020 were divided into groups 1–3 according to their serum fTLI concentration. Groups 1.1 and 2.1 represent animals with known clinical data. Group 2.1 included data from cats with serum fTLI concentrations in the lower reference interval (8–12 µg/L). In the next step, cats from groups 1.1 and 2.1 were divided into responsive (groups 1.1a and 2.1a) and non-responsive (groups 1.2b and 2.1b) to treatment with pancreatic enzymes.

Using the estimated reference interval, 275 (6%) of the 4813 cats displayed decreased serum fTLI concentrations <8 µg/L (group 1). Most of the cases (88%, *n* = 4256) had serum fTLI concentrations within the reference interval of 8–88 µg/L (group 2). Increased concentrations >88 µg/L were observed in 282 cats (6%, group 3). Further information, such as clinical signs and response to treatment, was requested from the submitting veterinarians for cats with decreased serum fTLI concentrations. Additionally, clinical data was enquired for cats in the lower reference interval of 8–12 µg/L to detect possible early-stage EPI. Follow-up examinations and samples submitted from other laboratories were excluded (*n* = 318). Partial clinical data from 200 cats could be obtained. The grouping is illustrated in Figure 2.

#### 3.2.1. Groups 1, 2, and 3: *n* = 4813

Data sets from 4813 cats were evaluated. The predominant breed of all cats was Domestic Shorthair (DSH, 63%, *n* = 3015) followed by mixed breed (5%, *n* = 233), British Shorthair (5%, *n* = 231), Maine Coon (5%, *n* = 219), Domestic Longhair (3%, *n* = 149), Bengal (3%, *n* = 132), Ragdoll (2%, *n* = 114), and Persian (2%, *n* = 100). In 58% of the sex of the cats was male (690 intact, 2098 castrated) and in 42% female (536 intact, 1489 spayed). The median age was ten years (range: 0.1–25).

**Figure 3:**
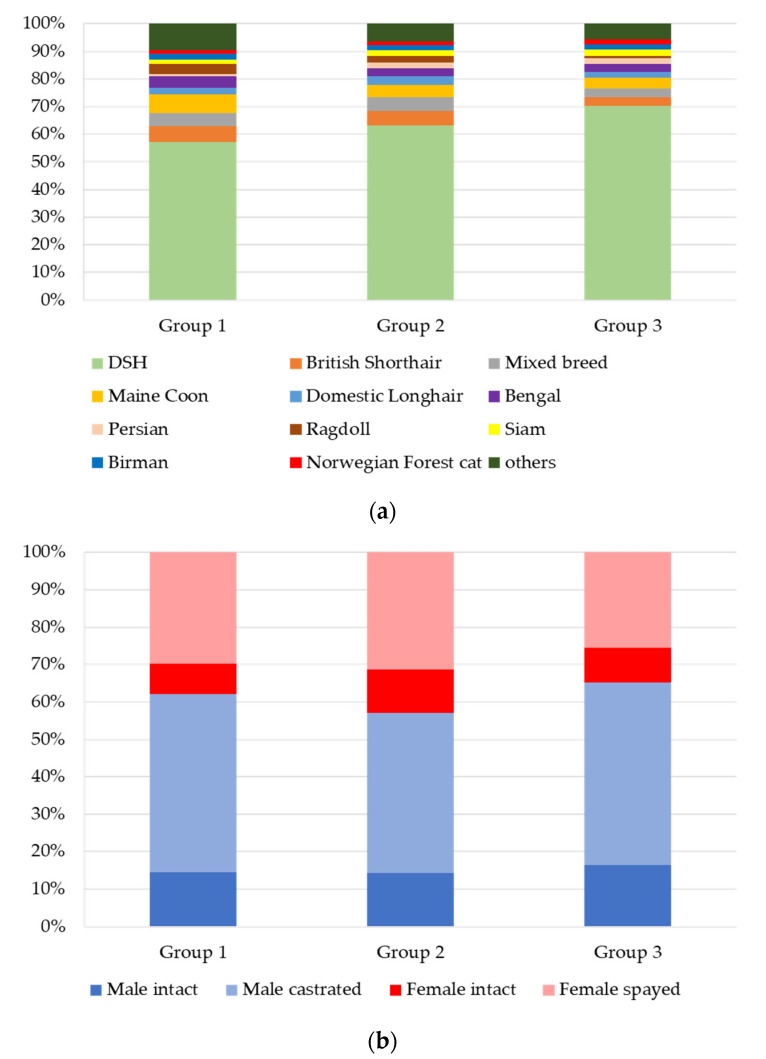

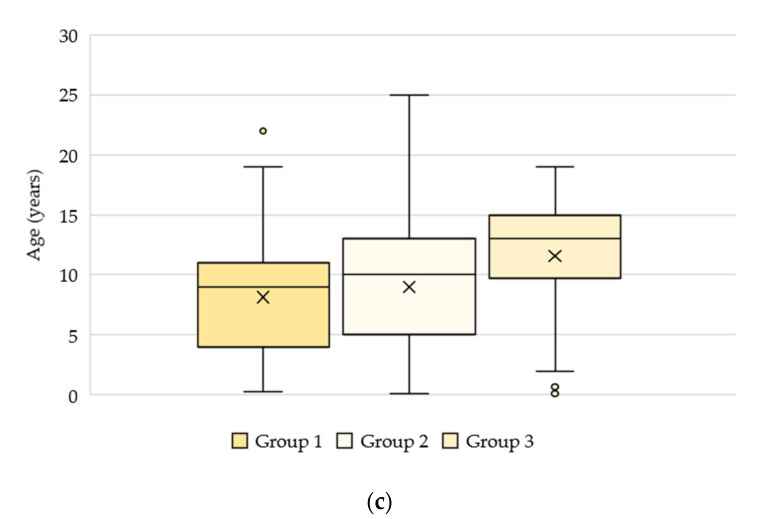
Comparison of the signalment from group 1 (fTLI decreased <8 µg/L), group 2 (fTLI reference interval 8–88 µg/L), and group 3 (fTLI increased >88 µg/L). (**a**): Breeds: The most common breed in all groups was Domestic Shorthair. The percentages of the other breeds were comparable in all three groups. (**b**): Sex: Percentages of intact or castrated males and intact or spayed females were similar in the groups. (**c**): Age: Cats with decreased serum fTLI concentrations had the lowest (nine years), and cats with increased serum fTLI concentrations had the highest median age (13 years). Outlier were included as circles.

When evaluating the groups separately with serum fTLI concentrations below (group 1), within (group 2), and above (group 3) the reference interval, Domestic Shorthair (DSH) was still the most common breed in all three groups and was reported in 57% (group 1, *n* = 157), 63% (group 2, *n* = 2660), and 70% (group 3, *n* = 198) of the cases (Figure 3a). The other breeds were also evenly distributed among the groups 1–3. Breeds frequently reported included Maine Coon (group 1: 7%, *n* = 19; group 2: 4%, *n* = 189; group 3: 4%, *n* = 11), British Shorthair (group 1: 6%, *n* = 16; group 2: 5%, *n* = 215; group 3: 3%, *n* = 9), mixed breed (group 1: 5%, *n* = 13; group 2: 5%, *n* = 211; group 3: 3%, *n* = 9), Bengal (group 1: 4%, *n* = 12; group 2: 3%, *n* = 112; group 3: 3%, *n* = 8), Ragdoll (group 1: 4%, *n* = 10; group 2: 2%, *n* = 102; group 3: 1%, *n* = 2), Domestic Longhair (group 1: 2%, *n* = 6; group 2: 3%, *n* = 137; group 3: 2%, *n* = 6), Birman (group 1: 2%, *n* = 6; group 2: 2%, *n* = 73; group 3: 2%, *n* = 5), and Persian (group 1: 1%, *n* = 2; group 2: 2%, *n* = 92; group 3: 2%, *n* = 6).

The sex distribution in the three groups was comparable (Figure 3b). Male cats were present in 62% (group 1), 57% (group 2), and 65% (group 3) and were more often castrated (group 1: 48%, *n* = 131; group 2: 43%, *n* = 1829; group 3: 49%, *n* = 138) than intact (group 1: 15%, *n* = 40; group 2: 14%, *n* = 604; group 3: 16%, *n* = 46). The distribution of female cats was 38% (group 1), 43% (group 2), and 35% (group 3). Female cats were either spayed (group 1: 30%, *n* = 82; group 2: 31%, *n* = 1335; group 3: 26%, *n* = 72) or intact (group 1: 8%, *n* = 22; group 2: 11%, *n* = 488; group 3: 9%, *n* = 26).

Median ages differed slightly between the groups. Cats with decreased serum fTLI concentrations had a median age of nine years (range: 0.3–22), while cats with concentrations in the reference interval displayed a median age of ten years (range: 0.1–25), and cats with increased serum fTLI concentrations had a median age of 13 years (range: 0.1–19) (Figure 3c).

#### 3.2.2. Group 1.1 (Serum fTLI <8 µg/L and Clinical Data Available): *n* = 87

Among the cats with decreased serum fTLI concentrations and clinical data available, Domestic Shorthair (52%, *n* = 45), British Shorthair (8%, *n* = 7), Maine Coon (7%, *n* = 6), mixed breed (7%, *n* = 6), Bengal (5%, *n* = 4), and Birman (5%, *n* = 4) were the most common breeds. The median age was eight years (range: 0.3–17), with more males (66%; 10 intact (12%), 47 castrated (54%)) and fewer females (34%; 6 intact (7%), 24 spayed (28%)).

**Figure 4:**
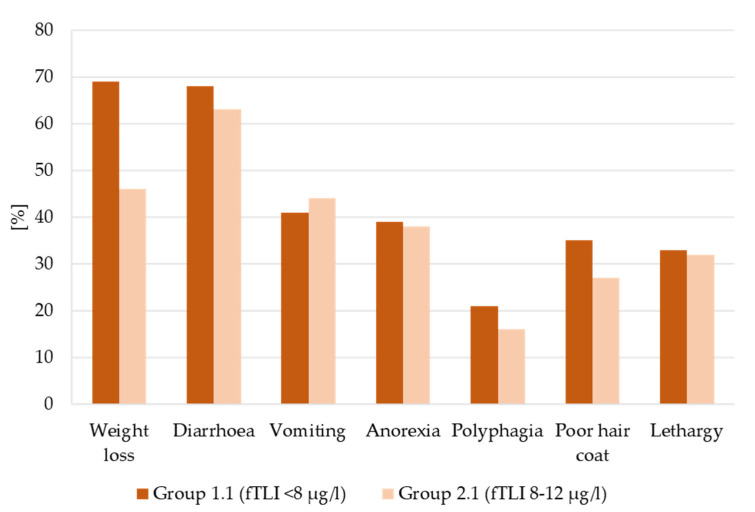
Percentage of occurrence of clinical signs in cats with decreased serum fTLI concentrations (group 1.1) and serum fTLI concentrations in the lower reference interval (group 2.1). Weight loss (group 1.1: 69%, group 2.1: 46%) and diarrhoea (group 1.1: 68%, group 2.1: 63%) were most reported in both groups, but cats with decreased serum fTLI concentration displayed more frequent weight loss. Other clinical signs were vomiting, anorexia, polyphagia, poor hair coat, and lethargy.

Clinical data for this group was available, but not necessarily complete for each case. Of the data available, weight loss (69%, 54 of 78 with documentation about weight loss) was reported as the most common clinical sign (Figure 4). Other gastrointestinal signs included diarrhoea (68%, 56/82), vomiting (41%, 34/82), anorexia (39%, 32/82), and/or polyphagia (21%, 17/80). Additionally, many cats also had poor hair coats (35%, 24/68) and/or lethargy (33%, 26/79). In 14 of 87 cases, only one clinical sign was reported (9× diarrhoea, 2× vomiting, 2× weight loss, 1× anorexia). The other cats had two (*n* = 24), three (*n* = 18), four (*n* = 13), or more than four (*n* = 14) clinical signs. Four cats suffered from various other clinical signs.

Information on response to treatment with pancreatic enzyme supplementation was available for 60 of 67 cases, treated with different enzyme extracts and variable posologies. Most of the cats (group 1.1a; 83%; *n* = 50) responded well to supplementation, while ten animals (group 1.1b; 17%) were documented as non-responsive. Of the non-responsive animals, two were euthanised shortly after blood sampling because of abdominal neoplasia (not further specified). One cat was diagnosed with inflammatory bowel disease (IBD), the remaining cats had an inconclusive diagnosis (*n* = 7). Almost half of the cats in group 1.1 (42/87) had very low serum fTLI concentrations <4 µg/L. Of this group, 34 animals received enzyme supplementation and in 31 of these cases, treatment response was documented. Most cats responded well to treatment (87%, 27/31) but four cats (13%) were “non-responsive”, which is comparable to the total group.

#### 3.2.3. Group 2.1 (Serum fTLI 8–12 µg/L and Clinical Data Available): *n* = 113

Clinical data was also evaluated from a subgroup of the cats with serum fTLI concentrations in the reference interval of group 2 (8–88 µg/L, group 2), to detect possible early-staged EPI. Cats with serum fTLI concentrations ranging from 8–12 µg/L were included. Like the other groups, the most common breeds included Domestic Shorthair (53%, *n* = 60), mixed breed (9%, *n* = 10), Maine Coon (7%, *n* = 8), British Shorthair (5%, *n* = 6), Bengal (4%, *n* = 4), and Norwegian Forest cat (4%, *n* = 4). In 55% of the cats the sex was male (13 intact (12%), 49 castrated (43%)), and in 45%, female (15 intact (13%), 36 spayed (32%)). The median age was eight years (range: 0.5–20).

Diarrhoea (63%, 66 of 105 with documentation about diarrhoea) was the most common clinical sign, followed by weight loss (46%, 46/101), vomiting (44%, 47/106), anorexia (38%, 37/97), lethargy (32%, 29/91), poor hair coat (27%, 21/77), and polyphagia (16%, 15/94) (Figure 4). The animals had one (*n* = 36), two (*n* = 24), three (*n* = 17), four (*n* = 12), or more than four clinical signs (*n* = 15). For nine cats, clinical signs other than those listed above were described.

Of the 40 cats receiving pancreatic enzyme supplementation (different enzyme extracts and posologies), 35 had information on treatment response available. In 66% (group 2.1a; *n* = 23), the animals had a good clinical response, while 34% (group 2.1b; *n* = 2) were “non-responsive”.

#### 3.2.4. Treatment Response in Groups 1.1 and 2.1 Combined

The diagnosis of EPI could be confirmed in 73 cats that responded to pancreatic enzyme supplementation. In 22 cats, no clinical improvement was observed and thus the EPI-diagnosis could not be verified by treatment. The signalment, clinical data, and serum fTLI concentration of cats which responded (groups 1.1a and 2.1a) and those that did not respond (groups 1.1b and 2.1b) to treatment are summarized in Table 1. The correlation between treatment response and serum fTLI concentration was statistically significant (*p* = 0.022). The lower the fTLI concentration, the more often cats responded well to treatment. However, there was no statistical significance with any of the other parameters, including sex, age, breed, weight loss, diarrhoea, vomiting, polyphagia, anorexia, poor hair coat, and lethargy (*p* > 0.05). Most of the treated cats were fed with a gastrointestinal diet and were already substituted with cobalamin. Thus, the cobalamin status of the cats was not considered.

## 4. Discussion

This study describes the signalment of cats for which samples were submitted for the measurement of serum fTLI concentration in a large study population of 4813 cats. Furthermore, for the assessment of the diagnostic utility of an in-house feline trypsin-like immunoreactivity (fTLI), selected clinical data of 200 cats with EPI defined by serum fTLI concentrations were selected retrospectively. Neither a breed nor a sex predilection for EPI could be observed in the examined cat population. A significant correlation was observed between a low fTLI and a good response to treatment with pancreatic enzymes, in cats with supposed EPI. Nevertheless, 66% of the cats with slightly decreased fTLI concentration demonstrated clinical improvement during treatment. Thus, like dogs, at least a repeated measurement of serum fTLI is recommended if the clinical signs and/or the clinicopathological parameters are inconclusive in cats.

There is no established gold standard for the diagnosis of EPI. Histopathology, which is often considered the gold standard for many other conditions, is not indicated if it is only pancreatic insufficiency that is suspected. For dogs, faecal elastase-1, which is a pancreatic protease, can also be assessed by ELISA [17]. Even though sensitivity of faecal elastase-1 is high (100%), specificity is not satisfactory (56.5%) [18]. Another study reported 23% false-positive results for faecal elastase-1 [19]. For cats, this test is not available. The current superior test for the diagnosis of pancreatic insufficiency in cats [1,6] and dogs [10] is serum TLI as a species-specific test. Definite numbers of sensitivity and specificity for serum fTLI ELISA were not published to the authors’ knowledge but is considered high [2]. However, severe protein malnutrition can cause decreased TLI because of an adaptive pancreatic response to low protein content in the diet [20]. Serum fTLI is not available as an in-house test for practitioners and must be submitted to a diagnostic laboratory. However, the associated higher turnover time can mostly be accepted because, generally, EPI is not an acute, life-threatening disease. As diagnostic therapy, a positive response to pancreatic enzyme supplementation may be indicative of EPI in cats as well.

According to Steiner [1], a breed predisposition has not been described for the feline EPI. In the previous study, the distribution of breed and sex in all groups (1–3) and subgroups (1.1–2.1) was similar. In all of them, Domestic Shorthair was the predominant breed, comparable to other studies [2,6,7]. The predominance of Domestic Shorthair is most likely a reflection of the common occurrence of this breed in the households in general and did not identify a breed predisposition. In contrast to this, a breed predisposition for canine EPI was described for German Shepherds, Chows, Cavalier King Charles Spaniels, and Rough-Coated Collies [21]. Even dog breeds that are underrepresented for EPI were described: Boxers, Golden Retrievers, Labrador Retrievers, Rottweilers, and Weimaraners [21].

All ages were represented in all groups but most of the cats were older (median ages 8–13 years). Cats with increased serum fTLI >88 µg/L (group 3) had the highest median age (13 years), while those with decreased serum fTLI <8 µg/L (group 1) had the lowest (nine years). Given the great disparity among groups, no statistical analysis was performed. For this reason, interpretation of the descriptive data must be done with caution. The cats for which clinical data were available (groups 1.2 and 2.1) had a median age of eight years, like other studies [2,6,7,22]. This is consistent with the assumed pathogenesis, as the most common cause for feline EPI is thought to be severe tissue destruction after one or multiple episodes of severe pancreatitis [6].

The cats in group 1.1 were more often male (66%) than female (34%), like in other studies [2,6,7,22]. However, when comparing them to animals with serum fTLI within the reference interval (group 2, *n* = 4256), there is no clear predisposition. In this group, males (57%) were also more often present than females (43%). This may suggest a greater number of male cats in the general feline pet population or higher susceptibility to illness in males rather than a male predisposition and no sex predilection for EPI in the cat population. In contrast, EPI in dogs mostly results from hypoplasia or pancreatic acinar atrophy [4].

The common clinical signs are non-specific and may overlap with many other diseases [1]. In the previous study, the main clinical symptom of cats with decreased serum fTLI (group 1.1) was weight loss (69%). Other studies reported higher percentages of weight loss in cats with EPI: 91% [7], 94% [2], and 95% [6]. Diarrhoea was reported in 68% of the cats with an fTLI value suggestive for EPI and 63% of the cases with an fTLI in the grey zone, following the literature (50% [6]–75% [2]). However, a direct comparison between these studies and our study is not possible as different methods for the detection of serum fTLI concentrations were used [9] and also because of variable criteria for the clinical diagnosis of EPI in these studies [2,6,7].

In our cohort, no statistical significance could be detected between responsive and non-responsive cats regarding signalment or clinical signs, following current literature [7]. Cats from group 2.1 (serum fTLI 8–12 µg/L) had similar clinical signs to those in group 1.1 (serum fTLI <8 µg/L), which underlines the fact that incipient EPI cats may be included in this group. This could be explained by the chronicity of the disease, as EPI manifests over time in this species, after presumably one to multiple episodes of pancreatitis [6].

In our study, 10/60 (17%) cats with serum fTLI <8 µg/L did not respond to treatment. Of these, two were euthanised because of neoplasia and one cat was diagnosed with IBD. For the remaining cats, a final diagnosis was not available. In two studies, all cats responded to enzyme supplementation treatment if the posologic guidelines were strictly followed [2,22]. However, the same number of studies found between 13% (16/121) [7] and 15% (3/20) [6] “non-responsive” animals, which is consistent with our data. The underlying reasons for a poor or a missing response are unclear, although several factors, such as a late-stage multimorbid patient, persistent pancreatitis, Diabetes mellitus, neoplasms, concurrent small intestinal diseases (IBD), other concurrent diseases (40–63% of the cats), and an insufficient treatment regimen are considered [2,6,7]. Pancreatitis is considered the most common cause of EPI in cats as discussed earlier and could still be persistent when EPI begins to manifest [6]. EPI together with Diabetes mellitus is seldom in cats but develop most likely secondary to destructive pancreatitis [2,6]. If one or more of these conditions were still present at the time of treatment, a solitary substitution of pancreatic enzymes may lead to mild improvement but not to a completely good general condition of the animal because further medication of the comorbidities is necessary. Thus, unknown concurrent disease could be an important consideration for non-responding to EPI therapy in our study as well. Also, cobalamin supplementation, as well as other medication, may be necessary for a good response [1,7,23]. Treatment with pancreatic enzymes is therefore recommended in cats with decreased fTLI concentrations after a complete workup of the case to exclude comorbidities or rather treat the other diseases adequately.

Our data demonstrated that the lower the fTLI concentration, the better the cats responded to treatment with pancreatic enzymes. This is consistent with another study where cats with a serum fTLI concentration <4 µg/L had a 3.2 times higher likelihood to respond well to treatment [7]. It may be explained by the progressiveness of this disease. The less functional pancreatic tissue is present, the more likely EPI is the main problem and treatment is probably effective.

Clinical improvement was observed in 23 of 35 (66%) cats with fTLI concentrations of 8–12 µg/L (group 2.1) and these cats were therefore considered to likely suffer from EPI. This suggests that serum fTLI concentrations in the lower reference interval should be assessed with caution, and it justifies a grey zone, as with the reference interval of the test so far, to increase sensitivity and detect these animals.

In dogs, repeated measurement of serum fTLI is recommended if the clinical signs and/or the clinicopathological parameters are inconclusive [24,25]. In a percentage of these dogs, the TLI value rebounded from decreased to normal when repeated [24]. In cats, similar studies are not available. Our data strongly suggest that animals with TLI concentrations in the lower reference interval may suffer from early-stage EPI or EPI with concurrent inflammation, and we recommend repeated measurements in this group, especially when EPI continues to be a suspected diagnosis.

A limitation of the previous study is the retrospective nature, as clinical data were obtained through the veterinarians’ medical reports, and the cats were treated with various kinds of enzymes and different posologic guidelines. Furthermore, the treatment response was subjectively evaluated by the clinician based on a binary system (yes or no) because no official, published grading system is available. Prospective studies including data collections over a longer period are needed to evaluate all factors that may influence the clinical course and treatment response and give a more differentiated picture. Detailed clinical pathology including kidney parameters would also be interesting for further studies. In dogs with decreased renal excretion during experimental acute kidney injury, TLI was reported to be within the reference range, even if an increase of this molecule was suspected because of a decreased relative filtration capacity [26]. In rats with chronic renal failure, TLI was increased in the pancreas but not in their serum [27]. In contrast, 2/28 dogs with renal failure in a control group of non-pancreatic diseases displayed increased fTLI concentration [28]. Thus, a simultaneous measurement of kidney values is recommended to better interpret the fTLI concentration.

The reference intervals were calculated using the Reference Limit Estimator, accredited by the Deutsche Gesellschaft für Klinische Chemie und Laboratoriumsmedizin e.V. [16], and verified the reference intervals previously established (<8 µg/L: EPI is likely, 8–12 µg/L: grey zone, 12–82 µg/L: reference interval; unpublished data). An important point for the use of the Reference Limit Estimator is animal welfare, as no animal-based studies are required for the calculation. With this tool, the animal’s health status is not necessary as it includes an algorithm that presumably excludes non-healthy individuals by decreasing the statistical error based on a high number of data sets [16]. This requires a minimum of 2000 data sets, with recommendations being 4000 data sets and above [16]. In our study, more than 4000 sets were included in both calculations. Reference intervals were first determined from 4813 data sets (2019–2020, 7–89 µg/L) and secondly from 21,714 data sets (2017–2020, 8–88 µg/L), concluding in an overall agreement. Alternatively, the American Society for Veterinary Clinical Pathology (ASVCP) suggests data sets of 120 and more healthy animals [29], for which sampling without any medical reason would be required. For another serum fTLI test, it was recommended that serum should be submitted after fasting for better results, which also applies for our test [30,31]

## 5. Conclusions

In conclusion, males and Domestic Shorthair cats were overrepresented in all groups, independent from serum fTLI concentrations. However, this probably represents the normal gender and breed tendencies of the domestic feline population, rather than a true predisposition. Clinical signs of cats with decreased serum fTLI concentration and of cats with values in the lower reference range were similar, but the first demonstrated weight loss (69%) more often than the latter (46%). The lower the serum fTLI concentration, the more often cats responded well to treatment with pancreatic enzymes. This stresses the importance of pancreatic enzyme supplementation in animals with low serum fTLI concentration. Additionally, more than half of the cats (57%) with serum fTLI concentrations in the lower reference interval (8–12 µg/L) also responded well to treatment. A follow-up examination and/or a therapeutic attempt should therefore be considered in such cats. fTLI is a helpful parameter for the diagnosis of EPI but predicting treatment response based on signalment or the clinical data included is not possible.

## Figures and Tables

**Table 1:** Statistical comparison of the signalment, clinical data, and serum fTLI concentration between cats which responded (*n* = 73) (groups 1.1a and 2.1a) and those that did not respond (*n* = 22) (groups 1.1b and 2.1b) to treatment.

Variable	Categories	Responder	Non-Responder	Total Number (*n*)	*p* Value
Sex	Male/female	39/34	14/8	95	0.400
	Male intact/male castrated/female intact/female spayed	8/31/10/24	1/13/2/6	95	0.708
Age (median)	Numbers in years	0.3–16 (7)	1–14 (9)	95	0.269
	<1 year/1–10 years/>10 years	5/45/22	0/14/8	95	0.221
Breed	DSH/others	34/39	15/7	95	0.077
Weight loss	Yes/no	43/21	14/6	84	0.815
Diarrhoea	Yes/no	49/20	12/8	89	0.353
Vomiting	Yes/no	33/35	8/14	90	0.322
Polyphagia	Yes/no	18/48	4/17	87	0.453
Anorexia	Yes/no	24/43	7/14	88	0.836
Poor hair coat	Yes/no	21/33	8/10	72	0.679
Lethargy	Yes/no	22/42	6/13	83	0.822
fTLI concentration (median)	Numbers in µg/L	0.09–11.8 (6.0)	0.09–11.6 (8.5)	95	0.022 *

DSH, Domestic Shorthair; fTLI, feline trypsin-like immunoreactivity; * *p* < 0.05.

## Data Availability

The data presented in this study is available in the article.
